# Emerging Role of ZBP1 in Z-RNA Sensing, Influenza Virus-Induced Cell Death, and Pulmonary Inflammation

**DOI:** 10.1128/mbio.00401-22

**Published:** 2022-05-19

**Authors:** Sharath Basavaraju, Sanchita Mishra, Rashi Jindal, Sannula Kesavardhana

**Affiliations:** a Department of Biochemistry, Indian Institute of Science, Bengaluru, Karnataka, India; The Ohio State; Albert Einstein College of Medicine

**Keywords:** influenza, inflammation, cell death, ZBP1, Z-RNA, SARS-CoV-2, innate immunity, inflammasome, RIPK3

## Abstract

Influenza viruses cause respiratory tract infections, which lead to human disease outbreaks and pandemics. Influenza A virus (IAV) circulates in diverse animal species, predominantly aquatic birds. This often results in the emergence of novel viral strains causing severe human disease upon zoonotic transmission. Innate immune sensing of the IAV infection promotes host cell death and inflammatory responses to confer antiviral host defense. Dysregulated respiratory epithelial cell death and excessive proinflammatory responses drive immunopathology in highly pathogenic influenza infections. Here, we discuss the critical mechanisms regulating IAV-induced cell death and proinflammatory responses. We further describe the essential role of the Z-form nucleic acid sensor ZBP1/DAI and RIPK3 in triggering apoptosis, necroptosis, and pyroptosis during IAV infection and their impact on host defense and pathogenicity *in vivo*. We also discuss the functional importance of ZBP1-RIPK3 signaling in recent severe acute respiratory syndrome coronavirus 2 (SARS-CoV-2) and other viral infections. Understanding these mechanisms of RNA virus-induced cytopathic and pathogenic inflammatory responses is crucial for targeting pathogenic lung infections and human respiratory illness.

## INTRODUCTION

Viruses have been coevolving along with their hosts to replicate and propagate as obligate intracellular parasites. Influenza viruses cause human respiratory infections, often emerging into epidemics and pandemics (1). Influenza virus contains a segmented RNA genome, which results in genome reassortments and the emergence of new variants (1, 2). Among four different influenza viruses that have been identified so far, influenza virus types A and B (IAV and IBV) cause severe respiratory illness in humans (1). Influenza is subtyped further based on its surface glycoproteins, hemagglutinin (HA), and neuraminidase (NA), which confer host tropism (3). The twentieth century has witnessed three pandemics caused by subtypes of IAV, the 1918 “Spanish” influenza, H1N1; the 1957 “Asian” influenza, H2N2; and the 1968 “Hong Kong” influenza, H3N2 (1, 2). The H1N1pdm09 strain of IAV caused the most recent pandemic. Aquatic birds are the natural reservoirs of influenza viruses that migrate to different geographical regions annually (1, 4). Sixteen HA and nine NA subtypes have been isolated from birds so far (3). Intriguingly, novel IAV-like viruses are isolated from bats with distinct HA and NA subtypes (5, 6). The reassortment capacity and interspecies transmission are the critical mechanisms responsible for causing seasonal disease outbreaks and pandemics.

IAV primarily infects human epithelial cells of the upper respiratory tract (URT). The epithelial cells throughout the respiratory tract are permissive to IAV infection (7, 8). IAV infection in URT is nonfatal, whereas the lower respiratory tract (LRT) infections seen in highly pathogenic influenza infections, like H5N1, are associated with increased morbidity and mortality (9). Host sensing of influenza infection triggers inflammatory signaling programs, leading to the extracellular release of cytokines and chemokines that orchestrate innate and adaptive immune responses (10, 11). Cytopathic effects due to virus replication and inflammatory cytokine release further promote inflammatory cell infiltration, leading to acute lung injury, alveolar edema, necrotizing bronchiolitis, and excessive bleeding (7, 10). This results in respiratory congestion and blunted gas exchange, clinically termed acute respiratory distress syndrome (ARDS) (12).

IAV infection triggers multiple types of programmed cell death (PCD) pathways in infected epithelial and immune cells *in vitro* and *in vivo* (8, 13). Host cell death primarily facilitates containment of viral spread, damaged tissue repair, and immune activation. However, uncontrolled or dysregulated cell death results in pathogenic inflammation and immunopathology in influenza-infected patients (8, 14).

Severe acute respiratory syndrome coronavirus 2 (SARS-CoV-2) infection causes coronavirus disease 2019 (COVID-19). Similar to IAV, SARS-CoV-2 causes respiratory tract infection, resulting in mild to severe respiratory disease in humans (14). It is crucial to understand the pathogenesis and host-virus interactions associated with pandemic-causing viruses for better preparedness for these outbreaks and to develop therapeutic interventions. This review describes the programmed cell death activation in influenza infection and how it facilitates antiviral host defense responses or lung inflammation leading to immunopathology. We further focus on Z-DNA binding protein 1 (ZBP1), an emerging innate immune sensor of viral Z-RNAs that regulate programmed cell death pathways and host defense responses during virus infections. The mechanisms of SARS-CoV-2-induced pathogenesis in the lungs are not clear. We discuss the possible role of programmed cell death, ZBP1, and its downstream effector, receptor-interacting protein kinase 3 (RIPK3), in SARS-CoV-2 and other viruses-induced epithelial cell death and inflammation.

### Influenza infection and lung pathogenesis.

The host tropism of IAV is determined by sialic acid (SA) receptors on host cells to which the viral hemagglutinin (HA) binds and the expression of proteases that cleave and activate HA (15, 16). Once influenza infects the respiratory epithelium, virus replication and shedding occur within 24 h of infection, which continues for 3 days. The viral titers decrease to nearly undetectable levels 6 to 7 days after infection (14). In severe influenza infections, virus replication and shedding persist for a prolonged period. This is associated with lung pneumonia and ARDS, which ultimately cause respiratory illness and death of the infected individual (7, 8).

Nonfatal influenza infections predominantly involve the URT and trachea. Fatal cases of influenza infection cause pneumonia, suggesting LRT infection (17, 18). The pattern of virus attachment varies along the respiratory tract; seasonal influenza viruses H1N1, H3N2, and other pandemic H1N1 viruses bind abundantly to the URT compared to the highly pathogenic avian influenza virus, H5N1 (19, 20). This could be one of the critical reasons for the high transmission rate seen in the former compared to that in the latter. H5N1 predominantly infects type II alveolar epithelial cells, alveolar macrophages, and nonciliated bronchiolar cells in the human LRT (18). Diffused epithelial sloughing, epithelial necrosis, and mononuclear inflammatory cell infiltration are the characteristic features during the acute stage of influenza infection (7). H5N1 infection also induces severe bronchoalveolar lesions and inflammatory responses at acute stages, which sustain several days after infection (21, 22). The influenza tropism in LRT partly explains the occurrence of severe pneumonia in pathogenic influenza infections. Increased neutrophil infiltration in the lungs is associated with epithelial necrosis in severe influenza infections (23). Notably, histopathological changes in the respiratory tract of pigs, ferrets, and mice are comparable with human influenza virus infection (24–26). The sustained pulmonary pathology in 1918 influenza infection correlates with aberrant activation of cell death and inflammatory pathways within 24 h of infection that persists for a prolonged time (27, 28). Thus, respiratory epithelial cell death or necrosis is a predominant clinical feature in influenza infection, and aberrant inflammatory responses contribute to severe respiratory illness.

### Pattern recognition receptor activation and host cell death in influenza infection.

Respiratory epithelial cells express and functionally operate several pattern recognition receptors (PRRs) as a host defense mechanism, which recognizes influenza components for promoting innate immune responses (29). PRR activation mounts type I/III interferon (IFN), proinflammatory cytokine, and chemokine responses to promote antiviral activity (29). Upon virus entry, the viral RNA genome, in the form of viral ribonucleoproteins (vRNPs), is delivered to the cytoplasm and transported to the nucleus, where viral genome replication and transcription occur (1, 29). The influenza viral RNAs are potential pathogen-associated molecular patterns (PAMPs) sensed by PRRs, retinoic acid-inducible gene I (RIG-I) and Toll-like receptor 3 (TLR3), to mount type I IFN signaling and proinflammatory cytokine expression (29–31). TLR7 also recognizes endosomal influenza RNAs in some immune cells to promote type I IFNs (29, 31, 32). Type I IFNs are secreted into the extracellular space that binds to the cell surface IFN alpha/beta receptor (IFNAR), resulting in the upregulation of interferon stimulated genes (ISGs), conferring an antiviral state.

Type I IFNs promote the expression of several genes associated with programmed cell death (PCD) activation, including ZBP1, mixed lineage kinase domain-like pseudokinase (MLKL), and tumor necrosis factor (TNF)-related apoptosis-inducing ligand (TRAIL) (33, 34). IAV infection activates an intracellular threat sensor, NOD-like receptor family pyrin domain containing 3 (NLRP3), in both epithelial and immune cells (13, 35–37). Upon activation, the NLRP3 recruits an adaptor protein, apoptosis-associated speck-like protein containing a CARD domain (ASC), to assemble into a large multiprotein signaling complex called the inflammasome (38). NLRP3 inflammasome promotes caspase-1 activation and the release of proinflammatory cytokines, interleukin-1β (IL-1β) and IL-18, and an inflammatory form of PCD called pyroptosis (39). Pyroptosis is a lytic form of inflammatory cell death driven by gasdermin family proteins (39–41). Activated caspase-1 cleaves gasdermin D (GSDMD) to liberate its N-terminal domain from the C-terminal autoinhibitory domain. GSDMD N-terminal domain forms pores on the membrane, allowing small molecule and water passage, leading to osmotic lysis and pyroptosis (39, 40). A recent study demonstrates that ninjurin-1 (NINJ1) is essential for plasma membrane rupture and pyroptotic cell death following GSDMD pore formation (42). Influenza-induced type I IFN signaling is essential for the NLRP3 inflammasome activation in primary macrophages, suggesting the interconnected host defense functions to efficiently restrict the initial viral spread (13, 36, 43). RIG-I and TLR signaling are essential for type I IFN signaling and programmed cell death in immune cells (macrophages). In contrast, RIG-I alone is sufficient to induce these host responses in nonimmune cells (lung fibroblasts) (34, 43). The dying infected cells release intracellular inflammatory stimulants and damage-associated molecular patterns (DAMPs) and recruit adaptive immune cells such as cytotoxic T lymphocytes (CTLs) (44, 45). CTLs act by directly killing virus-infected cells. An intriguing study shows that the loss of more than 10% of type I alveolar epithelial cells corelates with mortality of IAV-infected mice, demonstrating a threshold for epithelial integrity maintenance and proper gas-exchange function during lung infection (46). A balanced activation of the influenza-induced PCD restricts viral spread by eliminating cells that can become viral factories and mount tissue repair processes. However, excessive PCD of respiratory epithelial cells in response to influenza infection may result in severe immunopathology and irreversible lung damage, leading to mortality (8, 14). Thus, respiratory cell death during IAV infection can be a double-edged sword.

### Inflammatory cell death activation in influenza infection.

Influenza infection induces apoptosis, a noninflammatory form of programmed cell death (47, 48). Influenza-mediated activation of apoptosis-independent cell death pathways has been characterized only recently. Cellular necrosis was considered a passive cell death modality. A great number of recent studies establish the programmed activation of necrotic cell death (necroptosis and pyroptosis), which initiates a chain of inflammatory responses by releasing proinflammatory cytokines and DAMPs (39, 49, 50). Initial studies demonstrate that pharmacological inhibition of apoptotic caspase-8 (CASP8) results in TNF-induced necroptosis (51, 52). CASP8 inhibition promotes the assembly of a large amyloid-like signaling complex, called necrosome, which commits cell fate toward necroptosis (39, 49, 50). Host proteins with RIP-homotypic interaction motif (RHIM) play an essential role in the necrosome formation. The RHIM domain promotes homotypic protein-protein interactions between two RHIM-containing proteins. RIPK1, RIPK3, ZBP1, and TIR-domain containing adaptor protein inducing interferon-β (TRIF) are the only four human/mouse proteins with a RHIM domain (53, 54). TNF-mediated death-receptor signaling and IFN- and TLR-mediated innate immune signaling pathways instigate RHIM-protein-driven necrosome assembly (39, 49, 50). Under these conditions, RIPK3 is the critical mediator of necrosome formation and necroptosis activation.

**FIG 1:**
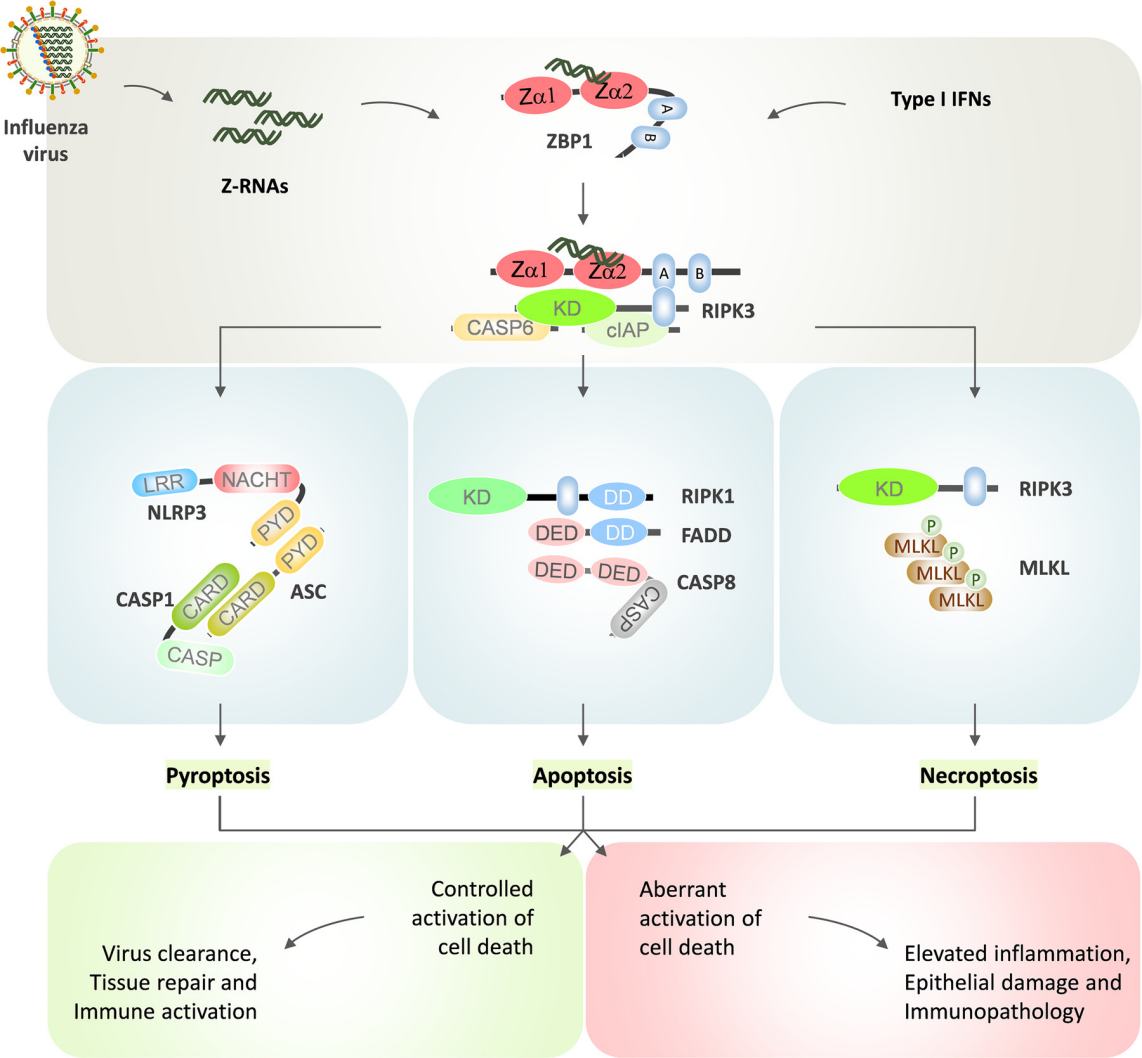
ZBP1 activation, cell death, and inflammation during IAV infection. IAV replication generates Z-RNAs and promotes type I IFN responses. IFN signaling upregulates ZBP1 expression. ZBP1 senses viral Z-RNAs, which triggers its activation and association with the RIPK3 through RHIM-mediated homotypic interactions. ZBP1 association with RIPK3 triggers activation of apoptosis and inflammatory cell death pathways. NLRP3 inflammasome activation promotes the release of proinflammatory cytokines (IL-1β and IL-18) and pyroptosis activation. RIPK3-driven phosphorylation of MLKL activates necroptosis and release of DAMPs. RIPK1-FADD-CASP8 complex triggers apoptosis activation. CASP6 facilitates RIPK3 and ZBP1 association to promote IAV-induced cell death and NLRP3 inflammasome activation. cIAPs also regulate RIPK3-mediated cell death and antiviral responses. Controlled activation of IAV-induced cell death is critical for antiviral responses, tissue repair, and immune activation. Dysregulated activation of cell death is associated with elevated inflammatory responses, epithelial damage, and immunopathology. In ZBP1 protein cartoon representation, A and B indications represent RHIM domains. CARD, caspase activation and recruitment domain; CASP, caspase; DD, death domain; DED, death effector domain; KD, kinase domain; LRR, leucine-rich repeat; PYD, pyrin domain.

In TNF-mediated death-receptor signaling, RIPK3 is known to associate with CASP8, Fas-associated death domain protein (FADD), and RIPK1 to form a cytosolic signaling complex named ripoptosome (55, 56). The ripoptosome, once formed, shows high plasticity in making cell fate decisions. This complex promotes CASP8-FADD-mediated apoptosis; however, loss of expression or inhibition of CASP8 and FADD results in a RIPK3-mediated necroptosis program (52). RIPK3 contains an N-terminal kinase domain and a C-terminal RHIM domain (39, 56). The RHIM-mediated RIPK3 interaction with RIPK1 or ZBP1 triggers assembly of the necrosome. The RIPK3 kinase activity facilitates MLKL phosphorylation, a crucial step for necroptosis execution (Fig. 1) (39, 49, 50). Phosphorylated MLKL oligomerizes and binds to phosphatidylinositol to form channel structures on the cellular membrane (57, 58). This results in an ionic imbalance and osmotic entry of water, causing cell swelling and eventually cell death. DNA viruses, such as herpesviruses (HSVs) and vaccinia virus, induce RIPK3-MLKL-mediated necroptosis in infected cells (59–61). This RIPK3-MLKL-driven necroptosis promotes antiviral and host defense responses in mice during herpesvirus infection (62, 63). However, these viruses have evolved to mimic host RHIM domains (RHIM decoys) as a host defense evasion strategy and restrict necroptosis to promote efficient viral replication and spread (59–62).

Unlike TNF receptor-mediated signaling, RIPK3 is activated without CASP8 blockade during IAV infection. IAV-mediated direct activation of the RIPK3 in lung epithelial cells is crucial for engaging both necroptosis and apoptosis (64). The earlier observations indicating predominant apoptosis activation in influenza-infected cells could be because of the usage of transformed human cell lines, like A549 and HeLa, which lack RIPK3 expression (47, 48). Recent studies demonstrate that IAV infection triggers multiple programmed cell death pathways in host cells (Fig. 1). Cellular inhibitor of apoptosis (cIAP) proteins regulate both apoptosis and necroptosis in host cells (65, 66). Loss of cIAP2 protein in mice triggers epithelial necrosis and degeneration in response to IAV infection (67). The absence of cIAP2 enhances mouse susceptibility and mortality to IAV infection due to severe bronchiolar degradation (67). Thus, there is a possibility that unleashing necroptosis by diverting apoptotic signaling (an indirect mechanism) triggers inflammation-driven immunopathology in IAV-infected mice. Lung necrotic pathology is a consistent feature in influenza patients with severe disease. Also, in lung tissue samples of patients with fatal ARDS-like H7N9 disease, cIAP2 expression seems to be significantly less (68), suggesting a protective role of cIAP2 in influenza infection through mitigating necroptosis.

Influenza infection also promotes NLRP3 inflammasome activation, which triggers pyroptosis of the infected cells, and the release of mature IL-1β and IL-18 (35, 37, 69, 70). IAV-induced NLRP3 inflammasome activation depends on upstream activation of the RIG-I and type I IFN signaling (36). Of note, transformed cell lines show the loss of ASC expression, which is essential for NLRP3 inflammasome formation (71).

### Role of RIPK3 in influenza infection.

The direct engagement of the RIPK3 activation in mouse fibroblasts and alveolar epithelial cells promotes apoptosis and necroptosis in response to IAV infection (64). A similar observation has been made using the human HT-29 cell line, indicating that RIPK3-mediated cell-death mechanisms are conserved between humans and mice (72). Studies using replication inhibitors and mRNA translation inhibitors revealed the requirement of IAV replication for activating RIPK3 cell death programs (64). RIPK3-deficient cells are resistant to IAV-induced cell death compared to wild-type (WT) cells, suggesting direct activation of the RIPK3 (34, 64). IAV infection and subsequent replication of its genome result in the formation of necrosome consisting of RIPK1, RIPK3, FADD, and MLKL, from which RIPK3 can either induce necroptosis or apoptosis (64). The mode of cell death is determined based on the stochastic availability of cell death components or the activity of RIPK3 or both (8). Apoptosis-promoting complex is essentially composed of RIPK3-RIPK1-FADD-CASP8, and the RIPK3-MLKL complex promotes necroptosis (73) (Fig. 1). Notably, the kinase activity of RIPK3 is critical for necroptosis, whereas RHIM domain interactions are crucial for apoptosis (8). Interestingly, IAV activates apoptosis and necroptosis redundantly, indicating the operation of compensatory defense mechanisms in host cells. Loss of MLKL results in a switch to apoptosis and deletion of the FADD results in necroptosis in IAV-infected cells (64). Remarkably, the combination of RIPK3 kinase and CASP8 inhibitors confers almost complete protection against IAV-induced cell death (34, 74). IAV induces necroptosis in cells in which CASP8 is active, challenging the notion that necroptosis is a backup form of cell death that appears only after caspase inhibition. Although the effect of apoptosis in restricting IAV infection *in vivo* was established, the necroptosis role remained ambiguous. IAV infection in knock-in mice expressing a CASP8 variant, which is nonfunctional to apoptosis but activates necroptosis, suggests that apoptosis and necroptosis are mutually exclusive defense pathways in IAV infection (74). This study also suggests that necroptosis activation can act as a stand-alone mechanism to clear IAV from infected lungs of mice (74). Even though RIPK3 deletion restricts IAV-induced PCD, apoptosis activation is noted at a late stage of infection in these cells (75). This indicates an alternative RIPK3 independent pathway promotes apoptosis activation, mediated by RIPK1, CASP8, and FADD signaling (34, 73, 75). RIPK3 activation and engagement of PCD pathways in response to IAV infection suggests upstream virus-sensing mechanisms in host cells for instructing RIPK3-mediated signaling programs.

### ZBP1, a central player in orchestrating IAV-induced programmed cell death and inflammation.

ZBP1 is a nucleic-acid binding protein with the RHIM domain, which was identified in tumor-associated tissues (76). ZBP1 was first implicated as a cytosolic DNA sensor promoting type I IFN signaling (77). RHIM domains of ZBP1 facilitate its interaction with other RHIM proteins like RIPK1 and RIPK3 *in vitro* (53, 54). Subsequent studies established a prominent role for ZBP1 in herpesvirus-induced necroptosis, making ZBP1 a potential interest because of its emerging role in innate immune sensing and its possible role in regulating cell death (59, 60, 62). Murine cytomegalovirus (MCMV) triggers ZBP1 activation, which complexes with RIPK3 to activate necroptosis (59, 78). Recent studies demonstrate an unexpected role of ZBP1 in sensing influenza infection to trigger multimodal PCD and proinflammatory cytokine release (34, 75) (Fig. 1). Genetic and biochemical studies suggest that ZBP1-deficient fibroblasts, respiratory epithelial cells, and macrophages are highly resistant to IAV-induced cell death (34, 75). These observations establish an essential role for ZBP1 in sensing influenza infection. Notably, ZBP1 association with RIPK3 assembles a cell death signaling complex to trigger apoptosis, necroptosis, and NLRP3 inflammasome-driven pyroptosis during IAV infection (34) (Fig. 1). In addition, ZBP1 expression in the IAV-infected epithelial lining of mouse lungs promotes cytotoxicity, and ablating its expression shows an intact epithelial layer (34, 75). Virus replication is critical for ZBP1 activation, and lack of ZBP1 expression does not ablate IAV replication. ZBP1 deletion confers complete resistance to IAV-induced cell death compared to RIPK3 deletion (34, 75). Deletion of RIPK3 and CASP8 or FADD and MLKL phenocopy ZBP1 deletion phenotype in IAV infection induced PCD (34, 75). RIPK1 acts as the adapter downstream of ZBP1 to recruit CASP8 via FADD and induce RIPK3-independent apoptosis during IAV infection. This unexpected but critical role of ZBP1 in IAV-induced cell death and inflammation suggests its role as an upstream activator of the RIPK3.

IAV-induced type I IFN signaling is essential for the upregulation of ZBP1 expression (34, 38, 43). Host cells lacking IFNAR1 expression are resistant to IAV-induced cell death and show reduced proinflammatory cytokine release, suggesting the upstream requirement of type I IFN signaling for upregulation of ZBP1 expression (Fig. 1) (38, 43). Although ZBP1 is critical for PCD activation in both immune and nonimmune cells, the upstream regulation of ZBP1 through type I IFNs is cell type specific. RIG-I-mediated type I IFN signaling promotes IAV-induced ZBP1 induction in nonimmune cells, whereas TLR and RIG-I signaling are required for ZBP1 induction in immune cells (43). IAV infection preferentially engages NLRP3 inflammasome activation (34, 37) (Fig. 1). ZBP1 is indispensable for CASP1 activation and the release of IL-1β and IL-18 in response to IAV infection, indicating the requirement of ZBP1 in NLRP3 inflammasome activation (34, 43). ZBP1-mediated CASP1 activation promotes GSDMD pore formation and pyroptosis (79). Both RIPK3 and CASP8 are necessary for ZBP1-mediated NLRP3 inflammasome activation (34). Based on these observations, ZBP1 is considered to induce a multifaceted cell death program called PANoptosis (pyroptosis, apoptosis, necroptosis) (79, 80). IAV-mediated ZBP1 activation in primary macrophages promotes the formation of a PANoptosome signaling complex consisting of critical components of pyroptosis, apoptosis, and necroptosis (80, 81). A recent study demonstrates an essential role of caspase-6 (CASP6) in ZBP1-mediated PANoptosis and inflammation (82). CASP6 facilitates ZBP1 and RIPK3 association and signaling complex formation during IAV infection (82). Owing to the central role of ZBP1 in activating IAV-mediated cell death and inflammation, it can be an attractive target for therapeutic intervention for modulating pathogenic immune responses.

### Z-RNAs as ligands for ZBP1 activation.

The emergence of ZBP1 in sensing influenza infection spurred its function in viral RNA sensing. ZBP1 contains N-terminal tandem nucleic acid binding Zα domains (Zα1 and Zα2) followed by RHIM domains and a functionally undefined C-terminal region (Fig. 1) (54, 83). Unlike other nucleic acid binding proteins, the Zα domains show unique specificity to Z-conformation nucleic acids (Z-DNA/Z-RNA) (13, 84). Deleting Zα domains or Zα2 alone restricts ZBP1-RIPK3 signaling-mediated PCD activation after IAV infection (75). Also, introducing point mutations at two specific amino acid residues (N122 and Y126) in the Zα2 domain, essential for Z-nucleic acid interaction, shows inhibition of IAV-induced PCD (75, 85). Endogenous deletion of the Zα2 domain of ZBP1 shows IAV induced cell death restriction and blunted NLRP3 inflammasome activation (79). These observations establish that IAV-RNA sensing by the Zα2 domain of the ZBP1 acts as an initiating event for IAV-induced PCD and inflammatory responses. ZBP1 binds to influenza RNAs generated during its replication, showing a striking similarity to RIG-I interaction with influenza RNAs (75). ZBP1-bound RNA pool represents subgenomic RNAs and are the viral ligands for ZBP1 activation (75). A recent study demonstrates the formation of influenza Z-RNAs and their localization with ZBP1 in the nucleus, providing substantial evidence of Z-RNA formation in infected cells and its sensing by ZBP1 (Fig. 1) (72). In addition, multiple recent studies show that endogenous retroviral RNAs, which attain Z-RNA conformation, act as ligands for ZBP1 activation, promoting sterile inflammation and embryonic lethality of mice (86–88). Overall, viral Z-RNAs are the *bona fide* ligands for ZBP1 activation to engage PCD and inflammatory responses. Adenosine deaminase acting on RNA 1 (ADAR1) is an RNA editing protein with Zα domains. Studies describing endogenous and viral Z-RNA sensing by ZBP1 and ADAR1 unraveled the critical role of Z-RNA sensing in physiological functions (89–91).

IAV replication and transcription occur in the nucleus. ZBP1 is primarily a cytoplasmic protein that translocates to the nucleus and colocalizes with influenza Z-RNAs following infection (72). Z-RNA sensing in the nucleus promotes MLKL activation and the disruption of the nuclear envelope and DNA leakage, which appear like “inside out” cell death (72). Absent in melanoma 2 (AIM2) protein is a DNA sensor that assembles inflammasome complex. IAV infection also triggers the AIM2 inflammasome activation at later stages (92). Perhaps, ZBP1-mediated nuclear envelope disruption and DNA leakage into the cytosol might promote AIM2 inflammasome activation during IAV infection (72, 92). Although nuclear translocation of ZBP1 is crucial for influenza-induced cell death, restricting its localization only to the cytoplasm does not inhibit cell death responses during IAV infection (72). These findings indicate that, in addition to sensing Z-RNAs in the nucleus, ZBP1 may sense influenza RNA transcripts or the genomic RNA in vRNP structures (which appear in Z-conformation) in the cytosol to engage PCD signaling.

### RHIM-function of ZBP1 in influenza induced cell death and inflammation.

ZBP1 interacts with RIPK1 and RIPK3 through RHIM-mediated interactions to activate NF-κB signaling and the downstream PCD pathways (13, 53, 54). IAV-induced ZBP1 activation engages RIPK1-dependent proinflammatory cytokine expression (TNF and IL-6) mainly through modulating ripoptosome (34). Compromising the RHIM function of ZBP1 abolishes IAV-induced PCD and inflammation (75). This suggests that both Zα and RHIM domains are essential for ZBP1 function during IAV infection. RIPK1 restricts spontaneous activation of ZBP1 and necroptosis in physiological conditions, the failure of which leads to embryonic lethality or inflammatory disease in mice (93, 94). This spontaneous ZBP1 activation occurs through recognizing endogenous retroviruses, and mutating the RHIM domain of RIPK1 triggers ZBP1 activation and embryonic lethality (13). Thus, the RHIM domain has evolved to control distinct cellular mechanisms of RIPK1, ZBP1, and RIPK3. ZBP1 is polyubiquitinated in response to IAV infection (43). The ubiquitination of RIPK1 and RIPK3 facilitates PCD signaling and inflammation. ZBP1 ubiquitination might have a regulatory role in IAV-induced cell death by facilitating signaling complex assembly. A recent study demonstrates that TRIM34 protein polyubiquitinates ZBP1 and regulates IAV-induced PCD (95).

### ZBP1 and RIPK3 signaling in IAV infection *in vivo*.

ZBP1- and RIPK3-mediated PCD activation confer host defense through destroying viral niches and promoting immune responses. IAV infection induces severe lung pathology and lethality in RIPK3-deficient mice compared to that in the wild-type (WT) mice (64). RIPK3-deficient mice also show higher viral loads in lung tissue owing to failure to clear virus-infected cells (64). Loss of ZBP1 expression confers high susceptibility, poor control of viral spread, and reduced survival rates after IAV infection in mice, suggesting the protective role of ZBP1 in influenza infection (75). *In vivo* studies using ZBP1- and RIPK3-deficient mice suggest the crucial role of PCD pathways, apoptosis, and necroptosis in restricting viral replication and spread and protecting the mice from IAV-induced lethality. Mice lacking MLKL expression, which are deficient in necroptosis activation, do not show increased susceptibility like WT mice, suggesting a compensatory role for apoptosis pathway in clearing virus-infected cells (72, 75).

Similarly, inactive CASP8-expressing mice, which are defective in IAV-induced apoptosis, show better viral clearance and less susceptibility to infection-driven lethality (74). Mice deficient in components of both apoptosis (FADD) and necroptosis (MLKL) pathway were highly susceptible to IAV infection (72, 75). This suggests that activating one of the PCDs, apoptosis or necroptosis, is necessary to mount antiviral immune responses and confer protection against IAV-induced respiratory pathology.

Aberrant cell death activation and excessive inflammation are associated with severe disease and lethality in pathogenic influenza infections. Is ZBP1-RIPK3 signaling-mediated PCD protective or detrimental in lethal influenza infections? MLKL-deficient mice do not confer lethality after modestly lethal doses of IAV (72, 75, 96, 97). When challenged with lethal doses of IAV, WT mice are highly susceptible to pulmonary disease. In lethal IAV infection, MLKL-driven loss of nuclear membrane integrity, necroptosis, and release of DAMPs trigger neutrophil recruitment to the lungs in WT mice, leading to severe disease (72, 96). Mice lacking MLKL show less severity and more survival after a lethal dose of IAV infection, suggesting the role of necroptosis in pathogenesis during severe influenza infections (72, 96). Significant differences in the outcome of pathology and lethality are reported based on the mode of virus administration into the respiratory tract. ZBP1 expression shows a protective role against intranasal IAV infection but elicits lethal effects when the IAV is infected intratracheally (98). Thus, ZBP1 likely promotes epithelial damage and inflammation during IAV infection *in vivo*. Furthermore, URT- and LRT-specific ZBP1 functions may determine protective or detrimental responses. Several studies demonstrate the *in vivo* role of ZBP1 in IAV-infected mice with varying outcomes. Infection studies using highly pathogenic influenza strains (H5N1 or H7N9) may facilitate the identification of the role of ZBP1 in host defense or disease pathogenesis.

NLRP3 inflammasome and CASP1 activation play an essential role in virus clearance and mounting tissue repair processes in IAV-infected mice (35, 37). Loss of NLRP3 or other inflammasome components confers high susceptibility to IAV-induced lethality in mice (35, 37, 69). However, NLRP3 inflammasome activation is detrimental in mice infected with highly pathogenic influenza, H7N9 (99). Exacerbated inflammatory responses elicited by CASP1 activation and release of IL-1 cytokines drive pathogenesis in H7N9 infection (99). This suggests that NLRP3 activation elicits antiviral responses to control viral infection but triggers excessive inflammation and severe disease during pathogenic influenza infections. In support of this, recent observations indicate that administration of NLRP3 inhibitor at the acute stage of IAV infection becomes detrimental to the host, but NLRP3 inhibitor administration at a later stage of the infection promotes better survival and recovery of the mice (100, 101).

### ZBP1-RIPK3 signaling in host-specific immune responses.

**FIG 2:**
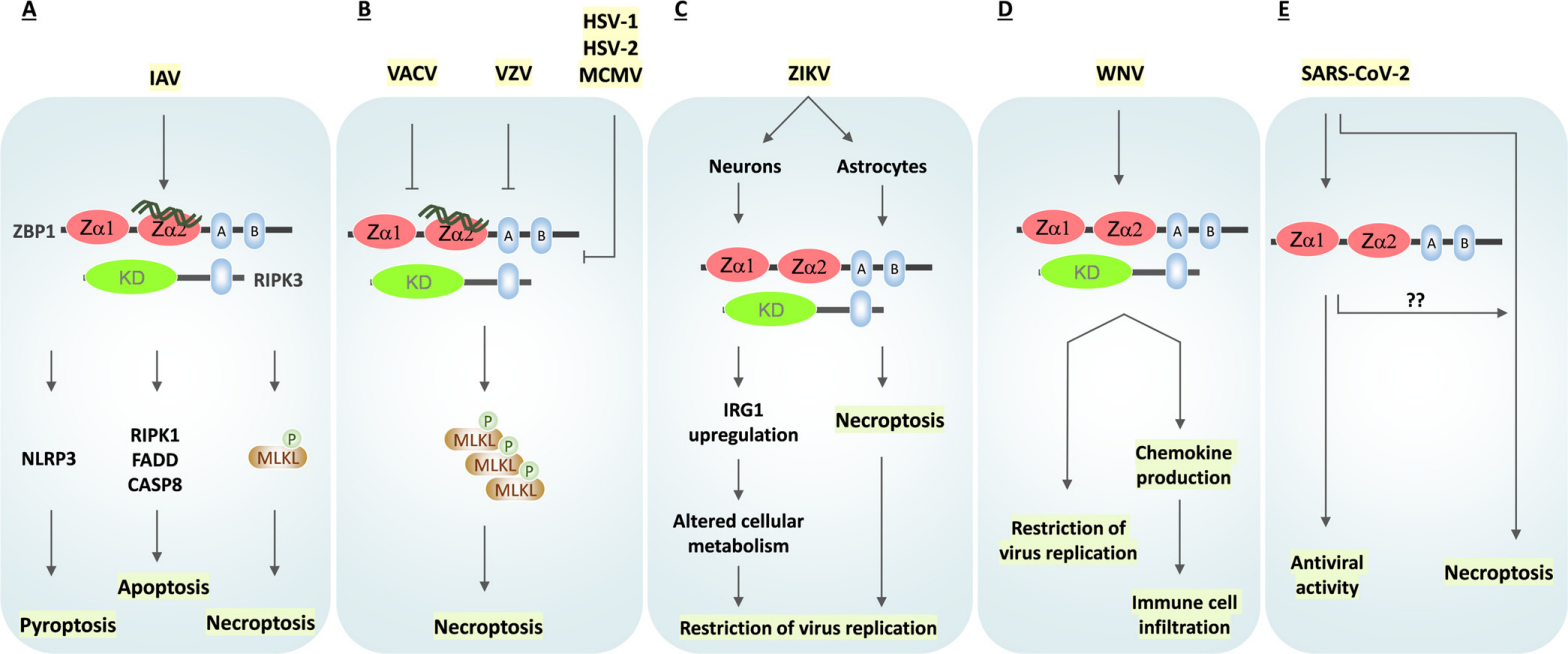
Role of ZBP1 in viral infections. (A) Influenza A virus (IAV) infection triggers ZBP1-mediated activation of apoptosis, necroptosis, and pyroptosis. (B) Murine cytomegalovirus (MCMV), herpes simplex virus 1(HSV-1), and HSV-2 express RHIM-decoy proteins (vIRA/M45, ICP6, and ICP10, respectively), which restrict ZBP1-RIPK3 signaling and necroptosis. Mutating these RHIM-decoy proteins trigger necroptosis activation and host defense. Varicella-zoster virus (VZV) also consists of a RHIM-decoy protein, ORF20, that forms complexes with ZBP1 to interfere with necroptosis activation. Vaccinia virus (VACV) E3 protein consists of a Zα domain protein that competes with ZBP1 for binding to Z-RNAs and inhibits necroptosis. (C) Zika virus (ZIKV) infection in neurons activates ZBP1-RIPK3 signaling, which promotes IRG1 upregulation. IRG1 further alters neuronal metabolism to facilitate the restriction of virus replication. ZIKV infection in astrocytes triggers ZBP1-mediated necroptosis that prevents viral spread. (D) ZBP1 is activated in West Nile virus (WNV) infection, promoting inflammation, immune cell infiltration, and inhibiting virus replication. (E) ZBP1 is associated with antiviral activity in SARS-CoV-2-infected cells. SARS-CoV-2 infection promotes necroptosis activation *in vitro*. Whether ZBP1 signaling activates SARS-CoV-2-induced necroptosis is not clear so far.

ZBP1-RIPK3 signaling is critical for IAV-induced PCD activation in mouse and human cells (Fig. 2A). The components of necroptotic cell death machinery are poorly conserved in the animal kingdom. ZBP1 first appeared in the amphibian class; RIPK3 is found only in the Craniata clade, while MLKL is found in Deuterostomes (102). However, the loss of these components is observed in specific classes during evolution. ZBP1 and RIPK3 are not found in the members of Aves class (birds), which are the reservoirs for influenza viruses (103, 104). These observations raise intriguing questions. Does the lack of necroptosis signaling components in birds contribute to viral tolerance? Could this absence of necroptosis help the virus replicate in the gastrointestinal tract of birds without triggering inflammatory immune responses? Whether the loss of ZBP1 and RIPK3 confers viral tolerance in birds needs further investigation. Birds show MLKL and RIPK1 expression, and perhaps the presence of other kinases may compensate for the loss of RIPK3 by directly activating MLKL (104). Also, MLKL retention in birds suggests the possible nonnecroptotic functions of MLKL. Nevertheless, it is still unclear whether necroptosis is active in birds that warrants further investigation. Conversely, necroptosis in humans may restrict the adaptation and spread of IAV among host species. Perhaps host-specific necroptosis activation may act as a bottleneck for zoonotically transmitted virus fitness and spread.

### Role of ZBP1 in other virus infections.

ZBP1 role in RIPK3 activation and necroptosis was demonstrated first in herpesviruses (105). MCMV vIRA protein (also called M45) consists of the RHIM domain that complexes with RIPK3 to inhibit necroptosis activation of infected cells (Fig. 2B) (59, 60). MCMV-mediated necroptosis inhibition confers high viral titers and spread *in vivo*. MCMV virus, in the absence of its RHIM-decoy protein vIRA/M45, activates ZBP1-RIPK3 signaling for triggering necroptosis (Fig. 2B) (59, 78). ICP6 and ICP10 proteins of HSV-1 and HSV-2 also have the RHIM domain that inhibits human RIPK3-mediated necroptosis activation (Fig. 2B) (62, 106, 107). Mutating ICP6 protein activates ZBP1-RIPK3 complex formation to promote necroptosis. Similar to MCMV and HSV-1, varicella-zoster virus (VZV)-encoded open reading frame 20 (ORF20) consists of an RHIM domain that complexes with ZBP1 (Fig. 2B) (108). VZV ORF20 restricts ZBP1-driven apoptosis in infected host cells. Recent studies demonstrate that vaccinia virus (VACV) interferes with ZBP1-RIPK3 signaling and necroptosis (61, 109). Both MCMV and VACV produce Z-RNAs in infected cells. ZBP1 interacts with MCMV viral RNA transcripts, suggesting its role in RNA sensing, which are in Z-conformation, to trigger cell death pathways (110, 111). An N-terminal domain deletion in the E3 protein of VACV promotes ZBP1 activation and necroptosis (Fig. 2B) (109). The N-terminus of the VACV E3 protein mimics Zα domain to compete with ZBP1 for binding to Z-RNAs (109). Thus, VACV E3 protein abolishes Z-RNA sensing by ZBP1 to restrict host cell necroptosis and host defense responses.

ZBP1 has also been implicated in Zika virus (ZIKV) infection (112), with distinct roles in infected neural cell types. Neurons show a distinct cell fate compared to astrocytes when infected with the ZIKV (Fig. 2C) (113). ZBP1 is upregulated in ZIKV-infected astrocytes, along with increased levels of phosphorylated RIPK1, RIPK3, and MLKL, culminating in necroptosis (113). However, ZIKV-infected astrocytes do not show signs of apoptosis and pyroptosis. Necroptosis activation not only clears virus-infected astrocytes but also helps in the release of cytokines and DAMPs that could contribute to the induction of innate and acquired immunity (Fig. 2C) (113). ZIKV accesses the central nervous system (CNS) through infecting neurons, but unlike astrocytes, ZBP1 activation in neurons does not show cell death activation. ZBP1-RIPK3 signaling does not engage necroptosis; instead, it upregulates immunoresponsive gene 1 (IRG1) protein, which alters the metabolic state of neurons to restrict viral replication (Fig. 2C) (114). Mice deficient in ZBP1 and RIPK3 show increased paralysis upon peripheral ZIKV infection and rapid mortality upon direct infection of the CNS, indicating the possible role of these necroptotic components (114). Similarly, in West Nile virus (WNV) infection, RIPK3 is indispensable for viral restriction by triggering chemokine production and mediating immune cell recruitment (Fig. 2D) (115). A recent study indicated that ZBP1 plays a critical role in controlling viral replication and also the viral loads upon WNV infection (116). These observations suggest that activation of ZBP1-RIPK3 signaling is triggered by several viruses, which promote PCD activation, inflammatory responses, or putative cell death-independent functions.

### Programmed cell death activation in SARS-CoV-2 infection.

The respiratory abnormalities in severe influenza and SARS-CoV-2 infection show several similarities (14). Respiratory epithelial damage and excessive cytokine responses have been demonstrated to cause ARDS in fatal cases of SARS-CoV-2 infection. Recent studies indicate apoptosis and necroptosis activation in SARS-CoV-2 infection *in vitro* (117–121). Studying postmortem lung sections of COVID-19 patients and lung tissues of SARS-CoV-2-infected nonhuman primates suggests apoptotic cell death activation in type 1 and type 2 alveolar epithelial cells and immune cells (119). Endothelial cells, which are nonpermissive to SARS-CoV-2, also show apoptotic signals (119). Recent reports provide clear evidence that virus-induced apoptosis contributes to lung damage and increases pathogenicity in SARS-CoV-2 infection (117, 119). High neutrophil infiltration is a common feature in severe SARS-CoV-2 infection, suggesting a possible role of necroptosis or pyroptosis in inflammatory responses (122). Coronavirus infection is demonstrated to engage PANoptosis activation and elevated cytokine responses (121, 123). Histological examination of neutrophil thrombi shows high levels of RIPK1, RIPK3, and MLKL in COVID-19 patients (124). These observations suggest SARS-CoV-2-mediated apoptosis and necroptosis activation in the human respiratory tract. However, necroptosis activation in SARS-CoV-2-infected respiratory epithelial cells and its functional relevance *in vivo* needs to be evaluated.

SARS-CoV-2 infection in CaLu-3 cells (human lung carcinoma cell line) induces CASP8-driven apoptosis and MLKL phosphorylation (118). A very recent study examined the impact of human interferon-stimulated genes (ISGs) on SARS-CoV-2 replication. This study indicates the antiviral activities of ZBP1 and MLKL during SARS-CoV-2 infection *in vitro* (Fig. 2E) (125). Also, ZBP1 promotes SARS-CoV-2-induced inflammatory cytokine production via the ZBP1-RIPK1-RIPK3 inflammatory signaling pathway (126). These observations suggest a potential role of ZBP1 in SARS-CoV-2-induced cell death and inflammation in human cell lines. However, whether SARS-CoV-2 infection generates Z-RNAs or activates ZBP1-RIPK3-mediated necroptosis is uncertain and needs further evaluation. Pangolins, in addition to bats, are hypothesized as a source of SARS-CoV-2 zoonotic transmission (127). Intriguingly, ZBP1 is inactivated by multiple in-frame stop codons in pangolins, which is defined as a pseudogene (128). Perhaps, ZBP1 might play a role in determining species-specific inflammatory responses against coronaviruses.

### Conclusions.

Innate immune and inflammatory responses are essential host responses to control influenza viral spread and mount tissue repair and protective immune responses. Excessive inflammatory cytokine responses and epithelial damage in the lung during pathogenic influenza infections suggest the dysregulation of cell death and inflammatory signaling pathways. Dampened inflammation is an essential mechanism that reservoir hosts show to tolerate pathogenic viruses without clinical manifestations. Thus, understanding the activation mechanisms of cell death and inflammatory responses is critical to define the specific pathways contributing to the elevated inflammatory responses and pulmonary immunopathology. ZBP1 has emerged as a crucial viral Z-RNA sensor to regulate cell death and inflammation during viral infections. Identifying the ZBP1-RIPK3 signaling axis in influenza-induced cell death facilitated the characterization of specific signaling pathways, which determine protective or pathogenic outcomes in influenza infections. Furthermore, loss of ZBP1 and RIPK3 in birds and functionally inactive forms of ZBP1 in pangolins indicate the critical role of these proteins in determining the pathogenicity of RNA viruses. Overall, investigating the specific mechanisms and proteins regulating PCD pathways and proinflammatory cytokine responses is crucial for understanding antiviral immune responses, immunopathology, and species-specific host responses.
